# Successful treatment of post-transplant relapsed acute myeloid leukemia with FLT3 internal tandem duplication using the combination of induction chemotherapy, donor lymphocyte infusion, sorafenib and azacitidine. Report of three cases

**DOI:** 10.1590/S1679-45082017RC3784

**Published:** 2017

**Authors:** Paulo Vidal Campregher, Vinicius Renan Pinto de Mattos, Marco Aurélio Salvino, Fabio Pires de Souza Santos, Nelson Hamerschlak

**Affiliations:** 1 Hospital Israelita Albert Einstein, São Paulo, SP, Brazil.; 2 Hospital São Rafael, Salvador, BA, Brazil.

**Keywords:** Leukemia, monocytic, acute/drug therapy, Induction chemotherapy, Antineoplastic agents, Recurrence, Case reports, Leucemia monocítica aguda/quimioterapia, Quimioterapia de indução, Antineoplásicos, Recidiva, Relatos de casos

## Abstract

Acute myeloid leukemia is a hematopoietic stem cell neoplastic disease associated with high morbidity and mortality. The presence of FLT3 internal tandem duplication mutations leads to high rates of relapse and decreased overall survival. Patients with FLT3 internal tandem duplication are normally treated with hematopoietic stem cell transplantation in first complete remission. Nevertheless, the incidence of post-transplant relapse is considerable in this group of patients, and the management of this clinical condition is challenging. The report describes the outcomes of patients with FLT3 internal tandem duplication positive acute myeloid leukemia who relapsed after allogeneic hematopoietic stem cell transplantation and were treated with the combination of re-induction chemotherapy, donor lymphocyte infusion, sorafenib and azacitidine. Three cases are described and all patients achieved prolonged complete remission with the combined therapy. The combination of induction chemotherapy followed by donor lymphocyte infusion, and the maintenance with azacitidine and sorafenib can be effective approaches in the treatment of post-hematopoietic stem cell transplant and relapsed FLT3 internal tandem duplication positive acute myeloid leukemia patients. This strategy should be further explored in the context of clinical trials.

## RESUMO

A leucemia mieloide aguda é uma doença neoplásica de células-tronco hematopoiéticas com alta morbimortalidade. A presença de mutações de duplicação em *tandem* de FLT3 leva a altas taxas de recorrência e a menor sobrevida global. Os pacientes com duplicação em *tandem* de FLT3 são normalmente tratados com transplante de células-tronco hematopoiéticas na primeira remissão completa. No entanto, a incidência de recidiva pós-transplante é considerável neste grupo de pacientes, e a conduta, nestes casos, é um desafio. O relato descreve os resultados do tratamento de pacientes com leucemia mieloide aguda positiva e duplicação em *tandem* de FLT3 que recidivaram depois do transplante alogênico de células-tronco hematopoiéticas e que foram tratados com combinação de quimioterapia de reindução, infusão de linfócitos de doador, sorafenib e azacitidina. São descritos três casos, e todos os pacientes apresentaram remissão completa prolongada com a terapia combinada. A combinação de quimioterapia de indução, seguida de infusão de linfócitos do doador, e a manutenção com azacitidina e sorafenib podem ser abordagens eficazes no tratamento da recorrência pós-transplante em pacientes com leucemia mieloide aguda e duplicação em *tandem* de FLT3. Essa estratégia deve ser mais explorada no contexto de ensaios clínicos.

## INTRODUCTION

While the majority of patients with acute myeloid leukemia (AML) will enter complete remission with induction chemotherapy, most will eventually experiment relapse of the disease. The only curative approach for AML patients after relapse is hematopoietic stem cell transplantation (HSCT). The risk of relapse can be estimated at diagnosis by the presence of cytogenetic alterations and gene mutations in the leukemic cells.^(1)^ AML patients with FLT3 gene internal tandem duplication (ITD) have a poor outcome, with a median overall survival of 13.1 months.^(2)^ Although AML patients with FLT3-ITD benefit from allogeneic HSCT in first complete remission (CR1),^(3)^ it has been shown that this subset of patients are at increased risk of relapse after HSCT.^(3,4)^

Post-transplant relapsed AML is associated with a high mortality rate. The use of chemotherapy followed by donor lymphocyte infusion (DLI) can provide long-term remission in less than 20% of patients.^(5)^ In addition, the use of azacitidine alone or in combination with DLI is associated with only 14% 1-year overall survival.^(6)^ Patients harboring FLT3-ITD mutations relapsing after HSCT can occasionally achieve sustained remissions with use of sorafenib, a tyrosine-kinase inhibitor, as monotherapy,^(7)^ but other investigators have reported sorafenib is not effective in this setting.^(8)^

We herein describe the outcomes of three patients with FLT3-ITD positive AML who relapsed after allogeneic HSCT, and were treated with the combination of re-induction chemotherapy, DLI, sorafenib and azacitidine.

## CASE REPORTS

### Case 1

A 49-year-old male was diagnosed with de novo AML with normal cytogenetics, positive for FLT3-ITD and nucleophosmin (NPM1) exon 12 mutations on November 2012. He was initially treated with one cycle of 7+3 and achieved a morphologic complete remission with presence of minimal residual disease by flow cytometry (0.1%).

He received one cycle of post-remission consolidation high dose cytarabine, and went on to receive a matched-related donor HSCT in first complete remission. Conditioning regimen consisted of busulfan plus fludarabine (BuFlu).

On day +54, he experienced leukemic relapse (62% blasts on bone marrow aspirate) and was salvaged with one cycle of bid cytarabine and fludarabine (fludarabine: 30mg/m^2^ q12 x 5d, cytarabine: 2,000mg/m^2^ q12 x 5d) followed by DLI (CD3: 1x10^7^/kg).

A bone marrow evaluation on D15 post-DLI revealed complete remission with 100% donor chimerism. Sorafenib was started immediately after DLI at a dose of 400mg twice daily. Therapy with azacitidine was started at 32mg/m^2^ per 5 days, once a month, beginning 1 month after DLI.

Side effects of sorafenib therapy included squamous syringometaplasia, successfully treated with topical corticosteroids, and gastrointestinal toxicity (diarrhea and abdominal pain) that was managed with intermittent sorafenib dose reductions. This patient developed grade I gastrointestinal and hepatic graft-*versus*-host disease (GVHD), 4 and 15 months after DLI, respectively, successfully managed with steroids. The last bone marrow evaluation was on August 2015 and the patient remained in complete remission with 100% donor chimerism, 28 months after DLI.

### Case 2

A 69-year-old male was diagnosed with de novo AML with diploid karyotype on May 2012. Molecular analysis revealed wild type FLT3 and the presence of NPM1 exon 12 mutation. The patient received induction therapy with 7+3, entered complete remission but developed severe infectious complications that led to impairment of his performance status.

He then received monthly consolidations with decitabine (20 mg/m^2^ D1-5) for 8 months, until February 2013, but relapsed 6 months later.

He was successfully salvaged with high-dose cytarabine and underwent a matched-related donor HSCT. Conditioning regimen was fludarabine and total body irradiation.

One year after transplantation, he had a second leukemia relapse. Molecular studies confirmed the presence of NPM1 mutation and a newly acquired FLT3-ITD mutation. The patient was salvaged with high dose cytarabine followed by DLI (CD3: 4.2x10^7^/kg) and sorafenib at 200mg twice daily.

Azacitidine was introduced at the dose of 32mg/m^2^ per 5 days, once a month, as maintenance after recovery of high-dose cytarabine. The patient had moderate gastrointestinal toxicity from sorafenib, and developed grade III gut GVHD 3 months after DLI, which was successfully treated with steroids.

Five months after DLI, a bone marrow aspirate revealed 1.6% blasts by flow cytometry that spontaneously disappeared during follow up. A bone marrow evaluation on July 2015 was consistent with complete remission with 100% donor chimerism, 17 months after DLI.

### Case 3

A 26-year-old female was diagnosed with de novo AML, in July 2013. The diagnostic workup did not include cytogenetics or molecular analysis. She was treated with 7+3, achieved a CR, and underwent matched-related donor bone marrow transplantation in September 2013.

Conditioning regimen consisted of BuFlu. She did not present any signs of GVHD. An evaluation on D84 revealed clear relapse with the presence of 80x10^9^/L blast cells in the peripheral blood.

Molecular analysis revealed the presence of homozygous FLT3-ITD. She received FLAG chemotherapy (fludarabine + high-dose cytarabine + granulocyte colony-stimulating factor, G-CSF) as salvage, but was found to be refractory. She was then treated with FLAMSA MEL (fludarabine, cytarabine, G-CSF, amsacrine) followed by infusion of CD34+ enriched DLI (CD3: 4.7x10^8^/kg).

Bone marrow evaluation on D30 post-DLI was consistent with complete remission. On D40 the patient developed grade IV acute gut and skin GVHD that was successfully treated with steroids and cyclosporine.

Tapering immunosuppression was started in March 2014 and finished in August 2014, when therapy with azacitidine (32mg/m^2^ per 5 days, once a month) was started.

Mild chronic skin GVHD, characterized by vitiligo, alopecia and mild scleroderma developed and was controlled with psoralen and ultraviolet light (PUVA) and topical steroids, without need of systemic immunosuppression. In November 2014, sorafenib was introduced at 800mg once a day, but the treatment was discontinued due to a severe skin reaction.

In January 2015, the patient presented a retrosternal positron emission tomography (PET-CT) positive tumor. Biopsy was consistent with a granulocytic sarcoma. Bone marrow evaluation revealed absence of disease and 100% donor chimerism. The patient was treated with radiation therapy followed by sorafenib 400mg a day. She remained in complete remission by bone marrow and PET-CT evaluation after 7 months of therapy on both sorafenib and azacitidine.

## DISCUSSION

Post-transplant relapse of AML is associated with a high mortality rate, chiefly if relapse occurs early after transplantation, with an estimated 2-year overall survival of 9% for patients relapsing before D100 after HSCT.^(9)^ Treatment attempts for achieving disease control have been re-induction chemotherapy, DLI, hypomethylating agents and targeted therapies. The success of these treatment modalities has been modest, and long-term survival has been achieved in less than 20% of patients.^(5,6,9)^ In the context of AML with FLT3-ITD, the use of sorafenib, a multi-kinase inhibitor with activity against mutant FLT3, has also been explored to treat post-transplantation relapse with conflicting results.^(7,8)^

This case report describes the outcome of three patients who achieved disease control after treatment of AML with FLT3-ITD post-transplantation relapse with the combination of chemotherapy, DLI, azacitidine and sorafenib. All three patients developed GVHD during treatment. It is worth mentioning that two out of three patients had early relapse (before D100), what is usually associated with a dismal prognosis,^(9)^ but both achieved durable complete remission.

The main objective during treatment of AML that relapses post-HSCT is the induction of a graft-*versus*-leukemia effect that is frequently accompanied by GVHD. The use of sorafenib induced anti-tumor immunity in a murine model, by decreasing the number of regulatory T cells and PD1+/CD8+ T cells in the tumor microenvironment,^(10)^ In addition, the use of sorafenib was more successful in the treatment of AML post-transplant relapse than pre-transplant relapse. This led to the hypothesis that the efficacy of sorafenib may depend on a synergism with allo-immune effects.^(7)^ Finally, in some series, the use of sorafenib was associated with the development or worsening of GVHD,^(11,12)^ suggesting the mechanism of action of sorafenib in inducing AML remissions may include direct effects in the leukemic blasts, as well as an immune-mediated effect.

The combination of azacitidine and DLI has been explored to treat post-transplantation AML relapse, but less than 20% of patients achieved durable disease control.^(6,13)^ Besides its direct effect in leukemic cells, azacitidine was shown to induce CD8+ T-cell responses against tumor antigens in AML patients, when given after allogeneic HSCT,^(14)^ suggesting that it may contribute to a graft *versus* leukemia effect. The combination of azacitidine and sorafenib was shown to induce complete remissions in around 27% of patients with pre-transplant relapsed AML, but the median duration of remissions was only 2.3 months,^(15)^ suggesting that this combination strategy is not effective in unselected AML patients relapsing before HSCT. We speculate that the combination of sorafenib and azacitidine may contribute to the induction of durable remission after post-HSCT relapse, through direct effects in AML blasts and by contributing to the graft-*versus*-leukemia effect.

## CONCLUSION

Although our case series is small, we provide evidence that the combination of azacitidine and sorafenib after chemotherapy and donor lymphocyte infusion can be an effective approach in the treatment of post-hematopoietic stem cell transplantation relapsed FLT3-gene internal tandem duplication-positive acute myeloid leukemia. This strategy should be further explored in the context of clinical trials.
